# Genome-Wide Identification of Wheat *WRKY* Gene Family Reveals That *TaWRKY75-A* Is Referred to Drought and Salt Resistances

**DOI:** 10.3389/fpls.2021.663118

**Published:** 2021-06-04

**Authors:** Hong Ye, Linyi Qiao, Haoyu Guo, Liping Guo, Fei Ren, Jianfang Bai, Yukun Wang

**Affiliations:** ^1^Beijing Engineering Research Center for Hybrid Wheat, Beijing Academy of Agriculture and Forestry Sciences, Beijing, China; ^2^College of Agriculture, Shanxi Agricultural University, Taiyuan, Shanxi, China; ^3^College of Horticulture, Northwest A&F University, Yangling, China; ^4^School of Agricultural Science and Engineering, Shaoguan University, Shaoguan, China; ^5^College of Life Science, Capital Normal University, Beijing, China; ^6^Division of Biological Science, Nara Institute of Science and Technology, Ikoma, Japan

**Keywords:** wheat, drought, salt, ectopic expression, transcriptome analysis, transcriptional profiling

## Abstract

It is well known that WRKY transcription factors play essential roles in plants’ response to diverse stress responses, especially to drought and salt stresses. However, a full comprehensive analysis of this family in wheat is still missing. Here we used *in silico* analysis and identified 124 *WRKY* genes, including 294 homeologous copies from a high-quality reference genome of wheat (*Triticum aestivum*). We also found that the *TaWRKY* gene family did not undergo gene duplication rather than gene loss during the evolutionary process. The *TaWRKY* family members displayed different expression profiles under several abiotic stresses, indicating their unique functions in the mediation of particular responses. Furthermore, *TaWRKY75-A* was highly induced after polyethylene glycol and salt treatments. The ectopic expression of *TaWRKY75-A* in *Arabidopsis* enhanced drought and salt tolerance. A comparative transcriptome analysis demonstrated that *TaWRKY75-A* integrated jasmonic acid biosynthetic pathway and other potential metabolic pathways to increase drought and salt resistances in transgenic *Arabidopsis*. Our study provides valuable insights into the *WRKY* family in wheat and will generate a useful genetic resource for improving wheat breeding.

## Introduction

It is well known that wheat is one of the most important and widely cultivated cereals, supporting 60% of the world’s population for their daily calorific and protein needs (Tadesse et al., 2017). The warranty of wheat production and safety thus has been the first-line mission for world development. However, the productivities of wheat in some regions are still very low due to various stress conditions such as drought, high salinity, heat, and cold. Drought and salinity are the major abiotic constraints for the yield of not only wheat but also other crops and will continue to act as major challenges to researchers and traditional crop breeders in the future (Bartels and Sunkar, 2005; Nezhadahmadi et al., 2013; Oyiga et al., 2016).

Plant response to environmental stress is regulated by complex signaling pathways and networks which are coordinated by transcriptional factors (TFs) (Jiang et al., 2015). WRKY TFs comprise a large gene family and are involved in various abiotic stress responses, including drought and high salinity stresses (Phukan et al., 2016). WRKY TFs encode proteins harboring a WRKYGQK amino acid sequence that can bind to W-box motif in the promoter for the regulation of target gene expression (Eulgem et al., 2000; Ciolkowski et al., 2008). The *WRKY* gene family is a large group that is composed of multiple members. The *Arabidopsis* and rice (*Oryza sativa*) genomes contain 75 *WRKY* genes (Riechmann and Ratcliffe, 2000) and 102 putative *WRKY* gene members (Wu et al., 2005), respectively. In *Arabidopsis*, the *WRKY* gene family is classified into three groups: group I (two WRKY domains), group II (one WRKY domain and C2H2 type of zinc finger motif), and group III (one WRKY domain and C2HC type of zinc finger motif) (Eulgem et al., 2000; Bakshi and Oelmüller, 2014). Group II can be further categorized into IIa, IIb, IIc, IId, and IIe subgroups based on the additional short conserved structural motifs (Eulgem et al., 2000; Chen et al., 2018).

The role of WRKY TFs in the mediation of drought and salt stresses has been reported in various plants. In *Arabidopsis*, *WRKY1*, *WRKY46*, *WRKY54*, *WRKY63*, and *WRKY70* contribute to drought stress tolerance *via* different signaling pathways (Ren et al., 2010; Qiao et al., 2016; Chen et al., 2017), while *WRKY8* and *WRKY75* regulate salt resistance (Chen et al., 2013; Hu et al., 2013). In rice, *WRKY47* and *WRKY80* regulate drought stress, *WRKY45* and *WRKY72* participate in salt stress, and *WRKY30* mediates both responses (Ricachenevsky et al., 2010; Yu et al., 2010; Tao et al., 2011; Shen et al., 2012; Scarpeci et al., 2013; Raineri et al., 2015). Besides these, the heterogeneous expression of maize *WRKY40* confers rice drought resistance in the transgenic plants (Wang et al., 2018).

In wheat, some *WRKY* genes have been reported to be involved in drought and salt stresses (Mondini et al., 2011, 2012). *TaWRKY1* and *TaWRKY33* are induced by drought stress, and the introduction of these genes in *Arabidopsis* confer the plants drought and/or heat resistance (He et al., 2016). Meanwhile, another wheat *WRKY1* gene (GenBank accession no. EU665424) mediates drought tolerance *via* an ABA-dependent pathway (Ding et al., 2016). Overexpression of wheat *WRKY2* enhances drought stress tolerance in transgenic wheat (Gao et al., 2018), while overexpression of wheat *WRKY44* and *WRKY46* enhances both drought and salt stress tolerances (Wang et al., 2015; Li et al., 2020). Besides this, two stress-responsive wheat *WRKY* genes, *WRKY2* and *WRKY19*, are confirmed to play roles in drought, salt, and freezing stresses (Niu et al., 2012). Although these studies provided information on the function of some of the individual *WRKY* gene members in wheat, the systematic and comprehensive information of the wheat WRKY gene family is still lacking, partially due to the complexity of the wheat genome (Appels et al., 2018).

In order to uncover the detail and to facilitate the future research on WRKY TF family in wheat, we performed a genome-wide identification of *WRKY* gene family members based on a conserved motif search from the recently released common wheat genome (Appels et al., 2018). In the current study, we present an overview of the gene number, classifications, and stress-induced expression patterns of wheat WRKY TF family members. We also demonstrate characteristics and molecular functions of *TaWRKY75-A* associated with drought and salt tolerance by analyzing the phenotype and RNA sequencing data sets of transgenic *Arabidopsis* expressing *TaWRKY75-A*. The results will serve as foundation to molecular study and genetic engineering for wheat against drought and salt stresses.

## Materials and Methods

### Identification and Phylogenetic Analyses of *WRKY* Genes in Wheat Reference Genome

The annotated coding sequences and protein sequences were downloaded from the International Wheat Genome Sequencing Consortium (IWGSC) website^1^. The seed file of the WRKY domain (PF03106) in Pfam 32.0 database^2^ (El-Gebali et al., 2019) was used as bait (perform hmmbuild program to establish HMM profile) to search against the local reference genome database by performing hmmsearch program *via* HMMER software (ver. 3.2.1)^3^ (Wheeler and Eddy, 2013). The threshold value was set as lower than 0.05. The gained proteins were then submitted to NCBI conserved domain search tool^4^ (Marchler-Bauer et al., 2015) and Swiss-Model online tool^5^ to further check the WRKY domain. All confirmed wheat WRKY protein sequences were used to carry out multiple sequence alignment using ClustalX 2.1 software (Larkin et al., 2007). The phylogenetic tree was established using MEGA 6.0 based on neighbor-joining (NJ) method with 1,000 bootstrap replicates (Tamura et al., 2011). Gene Structure Display Server^6^ was used to determine and visualize the structures of wheat *WRKY* genes (Hu et al., 2015). The Gene Structure Display Server Program^7^ was used to analyze the exon–intron structure of wheat *WRKY* genes. The MEME program was employed to identify conserved motifs in wheat WRKY proteins (Bailey et al., 2006).

### Naming of Wheat *WRKY* Genes

In order to name all wheat *WRKY* genes sequentially, we utilized a consistent naming pattern based on their phylogenetic relationships and chromosome locations in A, B, and D subgenomes (Bai et al., 2019). First, each name of the wheat *WRKY* genes was started with the abbreviation of the species name *Triticum aestivum* (*Ta*). Second, based on the phylogenetic relationships, genes that located in A, B, and D subgenomes and clustered into the same branch were thought as the homeologous triplets of one gene. The serial number of each *TaWRKY* gene was arranged as the order of wheat chromosome complement, i.e., from chromosome complement 1–7. Third, the serial number of *TaWRKY* gene in one chromosome complement was decided as the physical location and number of *TaWRKY* gene copies. *TaWRKY* genes, which located on the chromosome that contained the most copies, were numbered preferentially. If the number of copies was equal, the name priority was assigned to chromosome A. Finally, the names of *TaWRKY* genes included an A, B, or D, denoting their subgenome loci (Wang et al., 2017).

### Chromosome Locations and Classification of *TaWRKY* Family Members

Chromosome location information of *TaWRKY* genes was collected based on the genome annotation information. All *TaWRKY* genes were mapped to their respective locus in the wheat genome and visualized using shinyCircos (Yu et al., 2018). The classification of *TaWRKY* genes was performed through analyzing the phylogenetic relationship between *TaWRKY* genes and representative types (i.e., types I, IIa, IIb, IIc, IId, IIe, and III) of *Arabidopsis WRKY* genes. ClustalX 2.1 software and MEGA 6.0 were used to generate the NJ phylogenetic tree (1,000 bootstrap replicates).

### Expression Analysis of *TaWRKY* Family *in silico*

The expression data under different stresses, including drought, heat, cold, and osmosis, were downloaded from the Wheat Expression Browser (WEB)^8^ (Borrill et al., 2016; Ramírez-González et al., 2018). The transcripts per million values were log_2_-transformed to create a heat map by using WEB.

### Plant Materials and Growth Conditions

The wheat cultivar Chinese spring (CS) and *Arabidopsis thaliana* (Col-0) were used as plant materials in this study. The CS seedlings were planted hydroponically in a greenhouse with continuous light cycle at 25°C (Niu et al., 2012). *Arabidopsis* seeds were sterilized and sown on half Murashige and Skoog (MS) medium. These *Arabidopsis* seeds were then transferred to a growth chamber (continuous light cycle at 22°C with 50% humidity) after 3 days of freezing (4°C) treatment.

### Total RNA Extraction, Reverse Transcription PCR, and Quantitative Real-Time PCR

Total RNA was extracted using the RNA extraction kit (QIAGEN, Germany) following the manufacturer’s instructions. The genome DNA in RNA samples were digested using the RNAse-Free DNase Set (QIAGEN, Germany). The quality of the RNA samples was checked using NanoDrop 2000c (Thermo Fisher Scientific, United States) and gel electrophoresis. Reverse transcription PCR (RT-PCR) was performed using the PrimeScript^TM^ II 1st Strand cDNA Synthesis Kit (TaKaRa, Dalian, China). Quantitative real-time PCR (qRT-PCR) was carried out using SYBR Premix Ex Taq^TM^ II (TaKaRa, Dalian, China) on an ECO real-time PCR system (Illumina, United States). The reaction program was described previously (Wang et al., 2017). The *Arabidopsis ACTIN2* gene (AT3G18780) and wheat *18S-rRNA* gene were used as endogenous control. All qRT-PCR primers were designed using Primer Premier 5.0 software based on the subsequent coding sequence sets downloaded from IWGSC archive, v. 1.0, or TAIR^9^. The primers are listed in Supplementary Table 1. The relative expression levels of genes detected in this study were calculated using the comparative threshold cycle method 2^–ΔΔCT^ (Livak and Schmittgen, 2001). Each experiment was replicated three times.

### Cloning of *TaWRKY75-A* and Subcellular Localization

The coding sequence (CDS) of *TaWRKY75-A* was amplified using the special primers listed in Supplementary Table 1. The CDS of *TaWRKY75-A* was inserted into pENTR/D-TOPO vector (pENTR^TM^ Directional TOPO^®^ Cloning Kits, Thermo Fisher Scientific, United States) and confirmed by sequencing. The confirmed *TaWRKY75-A* CDS (no termination codon) was then inserted into the 16318GFP (pro35S:GFP) vector. Wheat mesophyll protoplast cell isolation and transformation were described previously (Li et al., 2016). Green fluorescent protein (GFP) signal was detected by laser confocal fluorescence microscopy (ZEISS LSM 880, Germany).

### Transcriptional Activation and Yeast One-Hybrid Assay

In order to test the transcriptional activity of *TaWRKY75-A*, the full-length coding sequence (222 amino acids) and interceptive sequences including 1–44 amino acids (N-terminal), 1–102 amino acids, 44–222 amino acids, and 102–222 amino acids (C-terminal) were cloned into the pGBKT7 vector, respectively. The empty pGBKT7 vector was used as the negative control. The generated constructs were transformed into the AH109 yeast strain. The transformants were diluted into different concentrations and selected on SD/-Trp and SD/-Trp/-His/-Ade/X-α-gal deficiency media. The transcriptional activities were evaluated according to their growth status at 30°C for 3 days in darkness.

To analyze TaWRKY75-A binding to W-box motif, yeast one-hybrid assay was performed. The promoter sequence of *Arabidopsis RD29A* gene (AT5G52310), which contains triple tandem repeats of the TTGAC cis-acting element, was cloned into the yeast expression vector pHis2.1 as the reporter construct (pHis2.1-W-box). The full-length coding sequence of *TaWRKY75-A* was cloned into the pGADT7 vector and fused with the GLA4 domain to generate the effector pGADT7- TaWRKY75-A. Both recombinant vectors were transformed into yeast strain Y187 and selected on SD/-Trp/-Leu plates at 30°C for 2 days. The surviving colonies were then transferred to SD/-Trp/-Leu/-His medium containing 35 mM 3-AT. The interaction between TaWRKY75-A and W-box element was evaluated by the transformants’ growth performance. All the primers used here are listed in Supplementary Table 1.

### Generation of Transgenic *Arabidopsis* Plants Overexpressing *TaWRKY75-A*

The coding sequence of *TaWRKY75-A* (no stop codon) was inserted into the pENTR/D-TOPO vector. Then, the gateway reaction was applied to pB7WG2 containing CaMV 35S promoter fused with GFP to generate *TaWRKY75-A*-pB7WG2 (Karimi et al., 2002). *Agrobacterium*-mediated transformation method was performed to generate the transgenic *Arabidopsis* lines (Zhang et al., 2006). Positive seedlings were selected by spraying Basta (1:1,000). Homozygous seedlings were established from T3 generation and used for the subsequent analyses.

### Stress Treatments and Expression Profiles of Selected *TaWRKY* Genes

For polyethylene glycol (PEG) and salt stress treatments of wheat, 2-week-old CS seedlings were watered with 5% polyethylene glycol 6000 (PEG6000) and 100 mM NaCl. The leaf tissues were then collected at 0, 1, 2, 6, and 12 h after treatments. The samples were frozen in liquid nitrogen immediately. The qRT-PCR primers were designed based on the conserved region of each *TaWRKY* gene (Wang et al., 2017) and are listed in Supplementary Table 1.

### Phenotype Observations and Physiological Index Measurements in Transgenic *Arabidopsis*

Germination rates and primary root growth assays of wild type (WT) and transgenic lines were performed as described previously (Li et al., 2016). For drought tolerance assay, 4-week-old plants of WT and transgenic lines grown in the soil mixture containing vermiculite and Metro-Mix were cultured without watering for 2 weeks and then were re-watered. The survival rates were calculated at 4 days after re-watering (Li et al., 2016). Relative water content in the leaves was measured using the previous method (Schonfeld et al., 1988). The chlorophyll content of leaf tissues was measured, according to the method described previously (Veronese et al., 2006), from the seedlings after 2 weeks of drought stress. For the salt tolerance assay, 4-week-old plants of WT and transgenic lines grown in soil mixture were watered with 100 mM NaCl continuously for 2 weeks. The survival rates were then calculated. The fresh weights of WT and transgenic lines under salt stress were measured from 50 1-week-old plants of WT and transgenic lines. All the experiments mentioned above were replicated five times.

### RNA-Seq Sample Collection and Sequencing

Three-week-old transgenic *Arabidopsis* plants (OE1 line) were cultured on plates containing half MS and then transferred in liquid medium containing half MS and 5% PEG6000 or 100 mM NaCl. The plants soaked in half MS solution were used as control. The samples of the control and treated groups were collected at 0, 2, 6, and 12 h after treatments. Total RNA extraction was carried out using the method mentioned above. Transcriptome library creation was created as described previously (Hu et al., 2016) and sequenced on Illumina Novaseq 6000 platform. All RNA-seq libraries had three biological replications, and 150-bp pair-ended reads were generated and used for further analyses.

### RNA-Seq Data Analysis and Gene Validations

The adapter sequences and low-quality sequences were removed using the NGS QC Toolkit (v 2.3.3) (Chen et al., 2015), and clean reads were mapped to the *Arabidopsis* reference genome sequences using TopHat (Kim et al., 2013). Differential expression analysis of two conditions/groups was performed using the DESeq R package (1.10.1). Genes with an adjusted *P*-value < 0.05 found by DESeq were assigned as differentially expressed. Genes with a fold change ≥1 and false discovery rate <0.05 were considered as differentially expressed genes (DEGs). The fragments per kilobase of transcript per million fragments mapped reads method (E et al., 2014) was used to calculate the expression abundances of genes. Gene Ontology (GO) enrichment analysis of DEGs was implemented by the GOseq R packages based on Wallenius non-central hyper-geometric distribution (Young et al., 2010). A Kyoto Encyclopedia of Genes and Genomes (KEGG) pathway analysis was performed (*P* ≤ 0.05) using BlastX searches against the KEGG pathway databas^10^.

### Statistical Analysis and Data Availability

One-way ANOVA followed by Tukey honest significant difference test was performed to detect differences as required. The RNA-seq data sets have been submitted to the National Genomics Data Center^11^ Genome Sequence Archive database under accession number CRA003799.

## Results

### Identification of *WRKY* Family Members in Wheat

**FIGURE 1:**
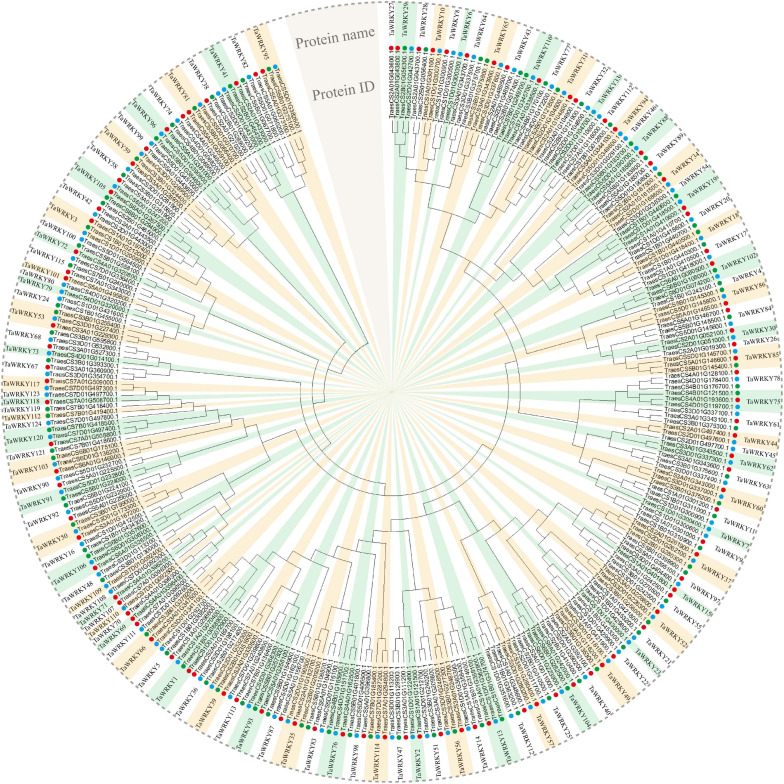
Phylogenetic analysis of 294 *TaWRKY* homeologous copies. An unrooted phylogenetic tree divides these homeologous copies into 124 *TaWRKY* genes. The neighbor-joining method is performed in MEGA 6.0 software. The protein ID in the International Wheat Genome Sequencing Consortium database and the protein name of each *TaWRKY* gene are shown. Red, green, and blue dots indicate *TaWRKY* homeologous copies from subgenome A, B, and D, respectively.

In order to examine the *WRKY* gene family in wheat, we searched the wheat genome using hmmsearch and WRKY domain seed file as query. In total, we obtained 353 protein sequences that possessed conservative WRKY domains (Supplementary Table 2). Among these candidate WRKY proteins, 47 proteins had two or more spliced isoforms (Supplementary Table 2), and for these the first isoform of each candidate WRKY protein was selected in the following analyses. After removing the spliced isoforms, a total of 294 WRKY coding sequences were confirmed (Supplementary Table 3). It is well known that common wheat is allohexaploid and contains A, B, and D subgenomes. Therefore, each wheat gene principally have three homeologous gene copies (Yu et al., 2020). Then, we generated an unrooted phylogenetic tree to confirm the homeologous gene copies of each *WRKY* gene. As shown in Figure 1, 294 gene copies, respectively, belonged to 124 *WRKY* genes (Figure 1). Because the *WRKY* gene nomenclature in wheat is currently not consistent, we renamed all genes based on their subgenome association (see “Materials and Methods” section for details). Then, we analyzed homeologous types of *TaWRKY* genes. It appears that over a half of these 294 *TaWRKY* genes have three homeologous copies (60.48%), and 16.14% of *TaWRKY* genes lost one homeolog (Table 1). Besides this, we found that 23.38% of the *TaWRKY* genes have only one gene copy, indicating that over one-fifth of the members are orphans/singletons (Table 1).

**TABLE 1:** Types of homeologous *WRKY* genes in wheat.

Homeologous type	Number of gene copies	Number of genes	% of genes^a^
A:B:D	225	75	60.48
A:B	2	1	0.81
A:D	24	12	9.68
B:D	14	7	5.65
A	10	10	8.06
B	4	4	3.23
D	15	15	12.09
Total	294	124	100

*^*a*^Percentage calculated with 124 genes.*

### Gene Structure, Conserved Domain, and Chromosomal Location

**FIGURE 2:**
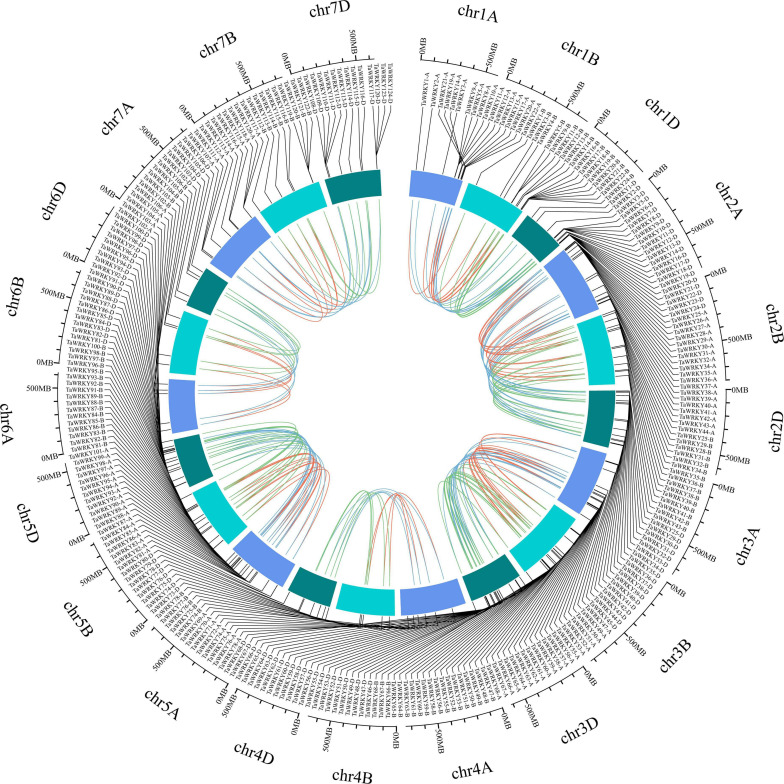
Chromosomal location of *TaWRKY* genes. All *TaWRKY* homeologous copies are mapped to their respective locus in wheat chromosomes in a circular diagram. Subgenomes are denoted by different shades of blue. Homeologous genes were inferred by phylogeny and linked with subgenome-specific colors: subgenome A, red; subgenome B, green; subgenome D, blue (inside of circle).

To gain further insights into the *TaWRKY* gene members, we surveyed the gene structure, conserved domain components, and chromosomal location of each *WRKY* gene copy. The *TaWRKY* genes showed a wide range in sequence lengths, with *TaWRKY76-D* having the longest (20.8 kb) and *TaWRKY2-D* having the shortest (0.5 kb) genomic sequence (Supplementary Figure 1). Besides this, the intron–exon structures of *TaWRKY* genes indicated normal distributions of introns and exons (Supplementary Figure 1). Most of the *TaWRKY* gene copies harbored two (58 gene copies) or three (139 gene copies) exons. Twenty-six gene copies had no intron, and 71 gene copies contained 4–7 exons (Supplementary Figure 1). Meanwhile, the conserved protein motifs in the TaWRKY family were predicted using the MEME tool. Ten types of motifs, which were named motif 1 to motif 10, were identified in each *TaWRKY* gene copy. The number of conserved motifs in each TaWRKY protein varied from 3 to 8; all members had motifs 1, 2, and 3, and most members had motifs 4, 5, and 6 (Supplementary Figure 2), indicating that the TaWRKY proteins are relatively conserved. Furthermore, we investigated the chromosomal locations of each of the 294 *TaWRKY* gene copies using the recently released IWGSC wheat genome. Chromosome 1 contained the highest number of *TaWRKY* genes (24 genes), and chromosome 6 had the lowest number of *TaWRKY* genes (five genes) (Figure 2 and Supplementary Figure 3). Meanwhile, we found that subgenome D had the highest number of *TaWRKY* gene copies (109; 37.07%), while subgenome B had the lowest number (87; 29.59%) (Figure 2).

### Classification of the *TaWRKY* Gene Family

**FIGURE 3:**
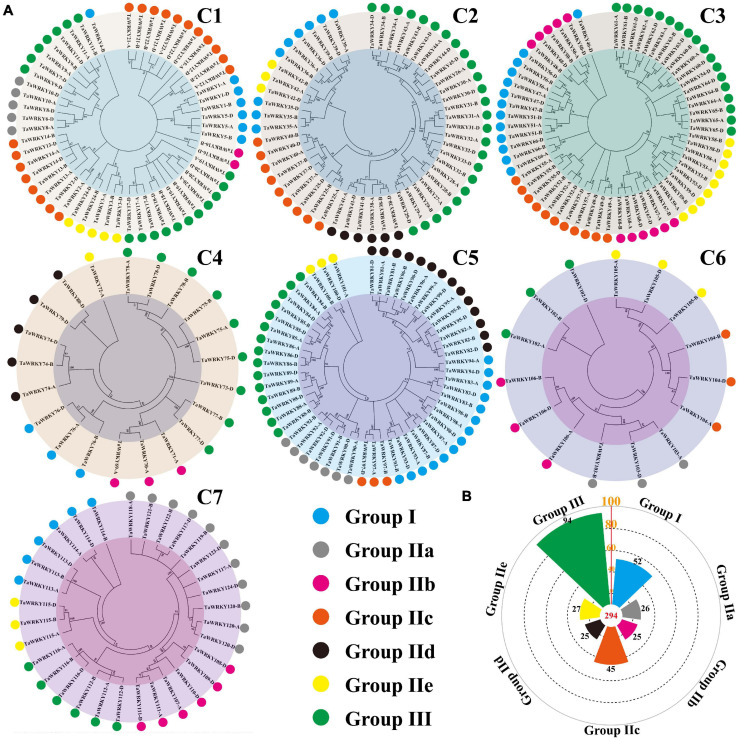
Phylogenetic classification **(A)** and number of TaWRKY homeologous protein in different groups **(B)**. Different colors show different WRKY groups. C1–C7 indicate the wheat chromosome clusters.

In *Arabidopsis*, the *WRKY* gene family is classified into three groups based on the number of WRKY domains and the types of zinc-finger-like motif (Eulgem et al., 2000). Among these groups, group II is further divided into subgroups from IIa to IIe (Eulgem et al., 2000; Bakshi and Oelmüller, 2014; Chen et al., 2018). To classify the *TaWRKY* gene members, we selected the typical *WRKY* genes of each group from *Arabidopsis* and performed the phylogenetic analysis with all *TaWRKY* genes (Supplementary Figure 4). Chromosomes 1 and 5 had six groups, and chromosomes 2, 3, 4, 6, and 7 contained five groups of *TaWRKY* members (Figure 3A). Among the 294 *TaWRKY* gene copies, 94 belonged to group III, which possessed a CCHC type of zinc finger motif (Figure 3B). A total of 52 gene copies were classified into group I, which had two WRKY domains (Figure 3B). The rest of the gene copies belonged to group II, which was separated into IIa, IIb, IIc, IId, and IIe subgroups and contained one WRKY domain and C2H2 type of zinc figure motif (Figure 3B).

### The Expression Pattern of the *TaWRKY* Genes Under Abiotic Stresses

In order to characterize the expression profiles of the *TaWRKY* gene family, we analyzed the RNA-seq data downloaded from expVIP (Borrill et al., 2016; Ramírez-González et al., 2018). We specially tracked the expression patterns of the *TaWRKY* family members after 2 weeks of cold (Li et al., 2015), 6 h of drought and heat (Liu et al., 2015), and 12 h of PEG treatments (Supplementary Table 4). Notably, there were 68 *TaWRKY* gene copies that could not be detected or that can be detected under only one of the cold, drought, heat, and PEG stresses (Supplementary Figure 5). The rest of the *TaWRKY* gene copies could respond to at least two types of stresses (Supplementary Figure 5). Under the drought and PEG treatments, *TaWRKY10*, *34*, *35*, *41*, *43*, *55*, *66*, *74*, *75*, *78*, *81*, *83*, *86*, *88*, *89*, *93*, and *112* displayed relatively high expression levels, and their gene copies showed similar expression trends under drought and PEG treatments (Supplementary Figure 5). Due to *TaWRKY10*, *34*, and *43* having been cloned and studied (Bahrini et al., 2011; Wang et al., 2013; Kumar et al., 2014; He et al., 2016), we excluded these three genes out of our later analyses.

**FIGURE 4:**
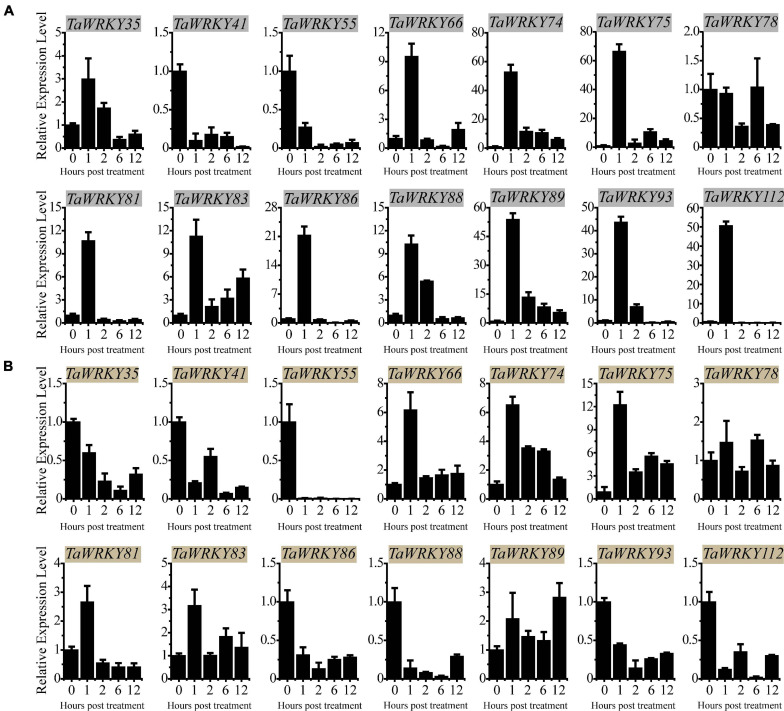
qRT-PCR assay-based expression patterns of 14 selected *TaWRKY* genes in 2-week-old wheat leaves under polyethylene glycol **(A)** and salt **(B)** stresses. All experiments were replicated three times, and the relative expression levels are normalized with wheat *18S-rRNA* gene. Bars indicate mean ± SD.

To investigate the expression profiles of the rest of the 14 *TaWRKY* genes, we monitored their expression trends under PEG (5% PEG6000) and high salinity (100 mM NaCl) stresses using qRT-PCR assay. Under PEG stress, *TaWRKY41* and *55* were down-regulated compared with mock (0 h) at each time point, and *TaWRKY78* remained almost unchanged (Figure 4A). The rest of the 11 *TaWRKY* genes responded to PEG stimulation after 1 h of treatment and displayed an increasing expression trends afterward (Figure 4A). Among these, *TaWRKY75* showed the highest expression level after 1 h of PEG treatment compared with the other members (Figure 4A). Under a high salinity stress condition, the expression levels of *TaWRKY35*, *41*, *55*, *86*, *88*, *93*, and *112* were inhibited at each time point (Figure 4B). No expression changes occurred in *TaWRKY78* as it did after PEG treatment. The expression of *TaWRKY81* increased after 1 h but was reduced subsequently compared to mock (0 h) (Figure 4B). Inversely, *TaWRKY66*, *74*, *75*, *83*, and *89* were induced at each time point compared with mock (0 h). Similar as in drought stress, *TaWRKY75* exhibited the highest fold change in the expression level at 1 h after treatment (Figure 4B). Notably, the physiological function of *TaWRKY75* has not been reported to date. Therefore, we hypothesized that *TaWRKY75* may serve as an important stress-related gene and conducted functional analyses as the following.

### Characterization of Transcription Activity of *TaWRKY75-A*

**FIGURE 5:**
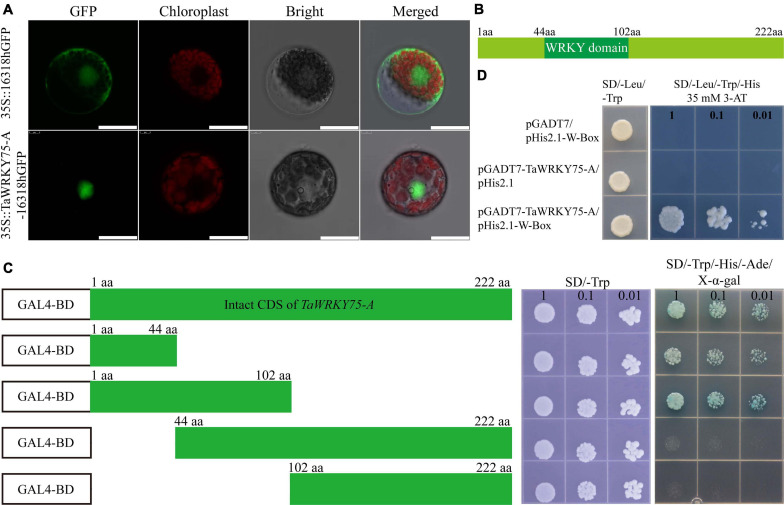
TaWRKY75-A is a typical WRKY transcription factor. **(A)** The subcellular location of *TaWRKY75-A*. Scale bars: 20 μm. **(B)** The protein structure of TaWRKY75-A. **(C)** Transactivation activity test of TaWRKY75-A in the yeast GAL4 system. CDS, coding sequence. **(D)** TaWRKY75-A can bind to the W-box motif. Yeast-one-hybrid assay was performed.

TaWRKY75 harbored one WRKY domain and a CCHC zinc finger motif, and the encoding gene had three copies on chromosome 4 (Figure 3A). To assess the molecular function of *TaWRKY75*, we cloned the nucleic acid sequence of one of its copies, designated *TaWRKY75-A*. *TaWRKY75-A* contained 669 base pairs and encoded the protein which had 222 amino acids. To observe subcellular localization, TaWRKY-A was fused with GFP under 35S promoter and transiently expressed in wheat protoplast cells. The TaWRKY75-A-GFP was detected in the nucleus (Figure 5A), indicating that TaWRKY75-A was a nucleus-localized protein. To evaluate the trans activity of *TaWRKY75-A*, the complete coding sequence and special fragments shown in Figures 5B,C were fused with the GAL4 DNA binding-domain coding sequence of pGBKT7 vector, and this recombinant construct was transformed into the yeast strain AH109. It showed that the N-terminal region without WRKY domain (1–44 aa) was minimally and sufficiently required for its trans activity (Figure 5C). It is well known that WRKY TFs bind the W-box sequence (C/T)TGAC(T/C) (Ulker and Somssich, 2004; Chen et al., 2018). Indeed the yeast-one-hybrid assay showed that TaWRKY75-A binds W-box with a conserved sequence (Figure 5D). Taken together, the results mentioned above showed that TaWRKY75-A was a typical WRKY transcription factor.

### Ectopic Expression of *TaWRKY75-A* Improved Drought and Salt Resistances in Transgenic *Arabidopsis*

The expression results indicated that *TaWRKY75-A* responded timely to drought and salinity stresses and pertained its high expression along continued stress input (Figures 1–4). To understand the function of *TaWRKY75-A* under drought and salinity stresses, we generated eight transgenic *Arabidopsis* lines by introducing *TaWRKY75-A* under constitutive 35S promoter and selected three lines (OE1, OE2, and OE3) for characterizing a drought- and salt-induced phenotype (Supplementary Figure 6).

**FIGURE 6:**
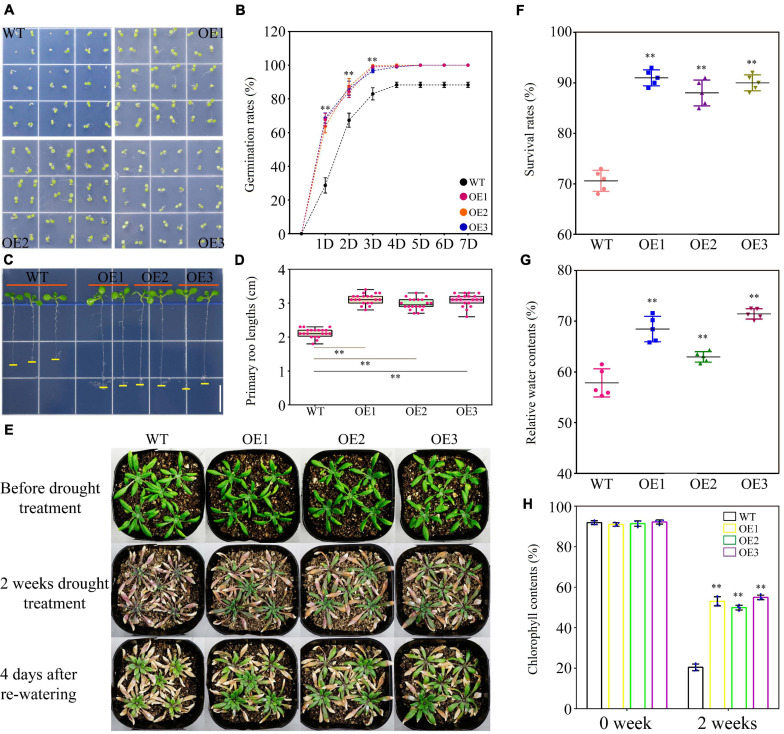
Overexpression of *TaWRKY75-A* enhances drought tolerance in transgenic *Arabidopsis*. **(A)** Observation of seed germination in wild type (WT) and OE lines at 4 days after germination. **(B)** Seed germination rate of WT and OE lines within a week. One-way ANOVA followed by Tukey honest significant difference (HSD) test was performed. ^∗∗^*p* < 0.01. Bars indicate means ± SD. This experiment was replicated five times. **(C)** Observation of the primary root elongation of WT and OE lines at 7 days after germination. Scale bar: 1 cm. **(D)** Statistics of lengths of the primary root of WT and OE lines at 7 days after germination. One-way ANOVA, followed by Tukey HSD test, was performed. ^∗∗^*p* < 0.01, *n* = 20. Bars show the maximum and minimum values. **(E)** Observations of WT and OE seedlings during drought test. **(F)** Statistics of the survival rate of WT and OE lines at 4 days after re-watering. One-way ANOVA, followed by Tukey HSD test, was performed. ^∗∗^*p* < 0.01. Bars indicate means ± SD. This experiment was performed five times. **(G)** Quantification of the relative water content of the leaves of WT and OE lines after 2 weeks of drought treatment. One-way ANOVA, followed by Tukey HSD test, was performed. ^∗∗^*p* < 0.01. Bars indicate means ± SD. This experiment was performed five times. **(H)** Statistics of the chlorophyll content of the leaves of WT and OE lines after 2 weeks of drought treatment. One-way ANOVA, followed by Tukey HSD test, was performed. ^∗∗^*p* < 0.01. Bars indicate means ± SD. This experiment was performed five times.

**FIGURE 7:**
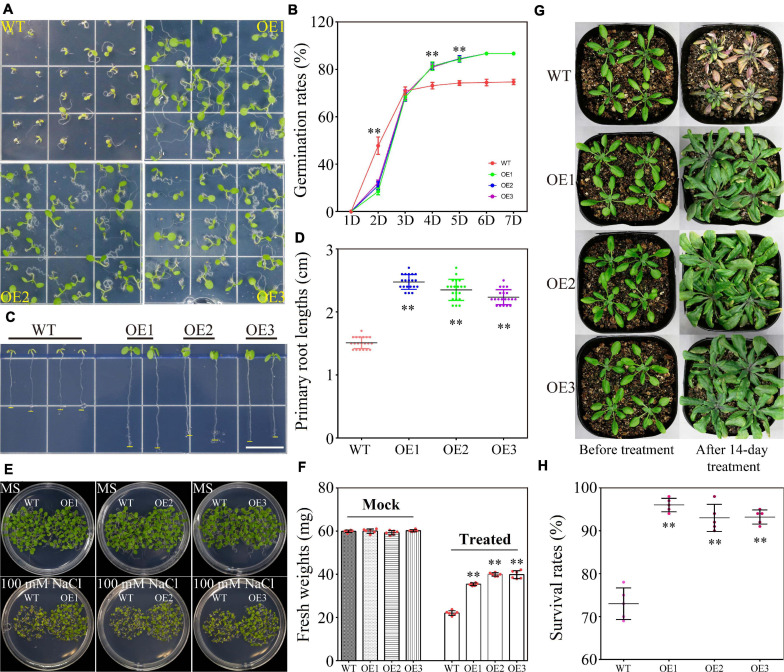
Overexpression of *TaWRKY75-A* increases salt tolerance in transgenic *Arabidopsis*. **(A)** Observations of seed germination in wild type (WT) and OE lines at 1 week after germination. **(B)** Seed germination rate of WT and OE lines within a week. One-way ANOVA followed by Tukey honest significant difference (HSD) test was performed. ^∗∗^*p* < 0.01. Bars indicate means ± SD. This experiment was replicated five times. **(C)** Observation of primary root elongation of WT and OE lines during 7 days after germination. Scale bar: 1 cm. **(D)** Quantification of primary root lengths of WT and OE lines at 7 days after germination. One-way ANOVA followed by Tukey HSD test was performed. ^∗∗^*p* < 0.01, *n* = 20. Bars indicate means ± SD. **(E)** Observations of the growing status of WT and OE seedlings under normal and salt conditions at 7 days after germination. **(F)** Quantification of fresh weight of 50 seedlings of WT and OE lines under normal and salt conditions at 7 days after germination. One-way ANOVA followed by Tukey HSD test was performed. ^∗∗^*p* < 0.01. This experiment was replicated five times. **(G)** Observations of WT and OE seedlings during salt stress. The salt stress was applied for 2 weeks. **(H)** Quantification of the survival rate of WT and OE lines after 2 weeks of salt treatment. One-way ANOVA followed by Tukey HSD test was performed. ^∗∗^*p* < 0.01. Bars indicate means ± SD. This experiment was performed five times.

No significant differences were observed under normal condition between the WT and transgenic *Arabidopsis* lines in seed germination rate and length of the primary roots (Supplementary Figure 7). PEG-induced stress greatly reduced WT germination within 7 days, while this suppressive effect was largely abrogated in the transgenic plants. The seed germination rates in WT and transgenic lines were eventually kept at 89 and 100%, respectively (Figures 6A,B). The primary root growth of WT was also inhibited in the presence of PEG and partially recovered in transgenic lines (Figures 6C,D and Supplementary Figure 7). Under a long-term drought stress condition (2 weeks without watering), the transgenic plants retained larger and greener leaves than those of WT. After re-watering treatment, the transgenic plants further exhibited better performance in survival rate, relative water content, and chlorophyll content than WT (Figures 6E–H). On the medium containing a high level of salt (100 mM NaCl), the seed germination rate of WT was reduced to 73%, whereas the transgenic plants germinated at a higher level up to 83% (Figures 7A,B). Salt also reduced primary root growth and fresh weights in WT but to a lesser extent in transgenic lines (Figures 7C–F and Supplementary Figure 7). Under a soil condition with long-term salt stress, the transgenic lines also showed a remarkably higher survival rate than the WT (Figures 7G,H). Our data altogether showed that the ectopic expression of *TaWRKY75-A* increased the drought and salt tolerances in *Arabidopsis*.

### Comparative Analysis of Transcriptome Data

**FIGURE 8:**
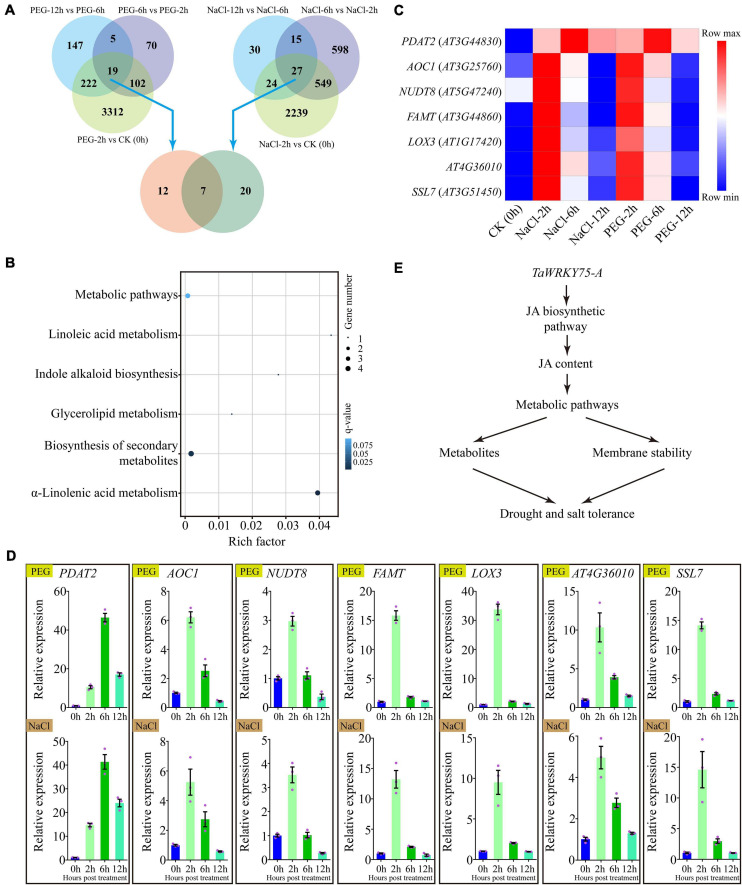
Comparative gene expression using RNA-seq in transgenic *Arabidopsis* overexpressing *TaWRKY75-A* after polyethylene glycol (PEG) and salt treatment. **(A)** Differentially expressed genes (DEGs) in *TaWRKY75-A Arabidopsis* after PEG or salt treatment compared across different time points of each treatment (top) for 0 (CK), 2, 6, and 12 h. A comparative analysis of DEGs between PEG and salt treatments was also conducted (bottom). **(B)** Kyoto Encyclopedia of Genes and Genomes pathway enrichment of seven common DEGs isolated from PEG and salt treatments in **(A)**. **(C)** Expression heat map of seven common DEGs after salt or PEG treatments. **(D)** qRT-PCR results of seven common DEGs after PEG (top) or NaCl (bottom) treatments. All experiments were replicated three times. Bars indicate means ± SD. The relative expression levels were normalized with *Arabidopsis ACTIN2* gene (AT3G18780). **(E)** The proposed working model of *TaWRKY75-A* regulating drought and salt tolerance. *TaWRKY75-A* can regulate the expression of jasmonic acid (JA) biosynthetic enzyme-encoded genes to change the JA content under drought and salt stresses. The downstream genes, which take part in several metabolic pathways, make a fast response to JA, resulting in the alterations of membrane stability and metabolites in cells. These alterations afford drought and salt tolerances in wheat and transgenic *Arabidopsis*.

To better understand the mechanism by which the ectopic expression of *TaWRKY75-A* increases drought and salt tolerances, we conducted the comparative transcriptome analysis in *TaWRKY75-A* transgenic plants treated with PEG or NaCl. A total of 3,655, 196, and 393 genes were differentially expressed at 2, 6, and 12 h after PEG treatment. Upon NaCl treatment, a total of 2,839, 1,189, and 96 genes were differentially expressed at 2, 6, and 12 h, respectively (Figure 8A). There were 19 and 27 genes that displayed expression changes throughout the PEG and salt-treated periods, respectively (Figure 8A). The GO and KEGG analyses of these DEGs indicated that they shared the same GO terms such as “membrane” and “membrane part” and KEGG pathways including “lipid metabolism” and “biosynthesis of other secondary metabolites” (Supplementary Figure 8), indicating that the ectopic expression of *TaWRKY75-A* might activate the same pathways *via* regulating the same genes to increase PEG and salt tolerance.

Furthermore, DEGs formed in PEG and salt treatments shared seven genes in common (Figure 8A). The GO function analysis results showed that six common DEGs participated in “metabolic process,” and five and four took part in “membrane” and “membrane part” terms, respectively (Supplementary Figure 9 and Supplementary Table 5). Meanwhile, the KEGG pathway analysis results revealed that three and four common DEGs were enriched in “α-linolenic acid metabolism” and “biosynthesis of secondary metabolites’ (*q*-value < 0.05), respectively (Figure 8B). Interestingly, these DEGs showed a similar expression pattern under PEG and NaCl treatments (Figure 8C), which was further confirmed by qRT-PCR assay (Figure 8D).

## Discussion

WRKY TFs, as a highly conserved protein family, are found in all plants and other eukaryotic lineages, such as fungi, Amoebozoa, diplomonads, and slime molds (Zhang and Wang, 2005; Liu et al., 2014; Rinerson et al., 2015). Progress in gene identification and functional evaluation of *WRKY* family members uncovered their important roles in plant adaptation to various environmental cues (Pan et al., 2017). In comparison to other crops, knowledge on *WRKY* gene family has remained relatively scarce in the wheat field due to the relatively backward release of complete and high-quality genome. Hence, a genome-wide systemic understanding of WRKY TFs would facilitate the evolution and function of particular *WRKY* genes in wheat development and stress response.

### Features of *TaWRKY* Gene Family During Evolution

In crop plants, the *WRKY* gene family has been well investigated. Until now, there are 103 and 119 *WRKY* genes that have been isolated in the rice (*O. sativa*) and maize (*Zea mays*) genome (Ramamoorthy et al., 2008; Wei et al., 2012), respectively. Our study revealed that the wheat genome possesses 124 *WRKY* genes. This number is similar as that of the rice and maize. Because wheat is hexaploid and has three subgenomes, the number of *TaWRKY* homeologous copies should be threefold of the *WRKY* genes in rice and maize in theory. We identified 294 *WRKY* homeologous gene copies in wheat, which is 2.85 and 2.47-folds compared to that in rice and maize, respectively. These indicate that the *TaWRKY* gene family is evolutionarily conserved in wheat in the context of gene number. The current and widely accepted classification system shows that the structures of *WRKY* genes classified in seven types are well defined in wheat and several other plants (Figure 3; Chen et al., 2018). In wheat, group III had the highest number of *WRKY* genes (Figure 3), which was similar to that in rice and maize (Ramamoorthy et al., 2008; Wei et al., 2012). This result suggested that the classification of *TaWRKY* is also conserved. Moreover, 10 different motifs are present in different TaWRKY proteins, with each protein comprising conserved motifs ranging from 3 to 8 motifs. In addition, all proteins have motifs 1, 2, and 3, demonstrating that the TaWRKY proteins have a conserved motif distribution profile.

As an allohexaploid plant, the percentage of wheat genes in homeologous groups for all configurations is highly similar across the three subgenomes: 63% (subgenome A), 61% (subgenome B), and 66% (subgenome D) (Appels et al., 2018). Among 294 *TaWRKY* gene copies, we found 109 copies (37.08%) on subgenome D, 87 copies (29.59%) on subgenome B, and 98 copies (33.33%) on subgenome A. These percentages were similar with the configurations of homeologous groups. Among 124 *TaWRKY* genes, 75 genes (60.48%) contained triads with a single gene copy per subgenome (an A:B:D configuration). Besides that, in common wheat genome, the three subgenomes exhibit similar levels of loss of individual homeologous gene, showing 10.7% (B:D), 10.3% (A:D), and 9.5% (A:B) of the homeologous types in the A, B, and D subgenomes, respectively (Appels et al., 2018). However, we found only one homeologous type that lost the homeolog on D subgenome (0.81%) in the *TaWRKY* family. Furthermore, we found that 10 (8.06%), four (3.23%), and 15 (12.09%) *TaWRKY* genes, which located on A, B, and D subgenome, respectively, belonged to orphan genes. These results demonstrated that the *TaWRKY* gene family did not undergo duplicate events other than gene loss during the long evolutionary process.

### *TaWRKY* Family Members Undertake Multiple Functions

Functional characterization of the individual *WRKY* gene in wheat has been carried out, primarily through interspecies gene expression. *TaWRKY2* (corresponding to *TaWRKY1* in our study) and *TaWRKY19* (corresponding to *TaWRKY35* in our study) are induced by salt, drought, and cold stresses. In *TaWRKY10* (corresponding to *TaWRKY47* in our study) transgenic tobacco, drought and salt stress tolerance is enhanced (Wang et al., 2013). Besides this, *TaWRKY45* (corresponding to *TaWRKY43* in our study) is upregulated in response to *Fusarium graminearum*, and the overexpression of *TaWRKY45* confers increased resistance against *F. graminearum* in transgenic wheat (Bahrini et al., 2011). It has been reported that *TaWRKY51* (corresponding to *TaWRKY38* in our study) negatively regulates ethylene production and then promotes lateral root formation (Hu et al., 2018). These evidence show us that different *TaWRKY* family members have different functions and also uncover the potential roles of *TaWRKY* genes in regulating abiotic and biotic resistances and developmental process.

Given that expression patterns can lead to estimation of gene functions (Xiao et al., 2018), we monitored the expression profiles of *TaWRKY* family members under PEG, cold, heat, and drought conditions. A total of 68 *TaWRKY* genes (54.84% of 124 *TaWRKY* genes) did not respond to these stresses, indicating that these genes do not respond to the relevant abiotic stress responses. A considerable number of *TaWRKY* genes (14 genes) are shown to respond to PEG and drought stresses from our analysis, highlighting that these genes are promising regulators for drought and salt responses in wheat. While more studies would be required for evaluating the physiological function of these individual genes, our study provides that core candidate genes can be targeted for improvement of wheat tolerance against drought and salt in the future.

### *TaWRKY75-A* Is the Positive Regulator of Drought and Salt Tolerance *via* Jasmonic Acid Biosynthesis and Membrane Metabolism

Lipoxygenase (LOX) and allene oxide cyclase (AOC) are two key enzymes that play decisive effects in jasmonic acid (JA) biosynthetic process (Farmaki et al., 2007). JA is one of the important phytohormones that regulate a plant’s response to abiotic stresses (Wang J. et al., 2020), and several JA-related genes including *JAZ*, *AOS1*, *AOC*, *LOX*, and *COI* have been reported to be upregulated upon various stresses (Robson et al., 2010; Hu et al., 2017). Drought condition also upregulates several JA biosynthesis and metabolism-related genes in wheat (Wang X. et al., 2020), and the exogenous application of JA was shown to enhance the activities of peroxidase, which, in turn, increases salt tolerance (Qiu et al., 2014). In addition, *TaAOC1*, which encodes an allene oxide cyclase involved in the a-linolenic acid metabolism pathway, has been demonstrated to enhance salt tolerance in wheat (Zhao et al., 2014). Our study reveals that *TaWRKY75-A*, when ectopically expressed in *Arabidopsis*, enhances drought and salt tolerance, most likely by upregulating JA biosynthetic genes *AtLOX3* and *AtAOC1*, and this mechanism may be conserved in *Arabidopsis* and wheat.

In addition to JA-related genes, we found that a lecithin/cholesterol acyltransferase family protein, PDAT2, a calcium-dependent phosphotriesterase superfamily protein, SSL7, and an undefined gene *AT4G36010*, which are all clustered into “membrane” and “membrane part” GO terms, are upregulated under PEG and salt stresses in *TaWRKY75-A* transgenic *Arabidopsis*. These results indicate that *TaWRKY75-A* might enhance drought and salt resistance by altering cell membrane homeostasis *via* these genes. Besides that, we found that a nudix family protein NUDT8 and a farnesoic acid carboxyl methyltransferase (FAMT) are induced after PEG and salt treatments. In the JA biosynthesis defective mutant *aos*, the expression of *NUDT8* is decreased significantly (Yan et al., 2007), suggesting that JA is required for *NUDT8* expression in *Arabidopsis*. The expression of FAMT is promoted when exogenous methyl jasmonate is applied (Yang et al., 2006), revealing that this gene is a downstream component of the JA signaling pathway. This is in line with the previous finding that lipid-derived hormone JA plays key functions on the direct regulation of plant metabolism (Savchenko et al., 2019). Thus, JA might regulate metabolic processes in our transgenic plants. Hence, based on these data, we propose that *TaWRKY75-A* is a key stress-resistant gene in wheat for adaptation to drought and salt stress by modulating the JA biosynthetic pathway as well as other metabolic pathways (Figure 8E).

In summary, our study provides comprehensive insights into the *WRKY* gene family in wheat. Classification of all TaWRKY TFs from whole genome survey will inevitably contribute to further molecular research for the identification and understanding of key *TaWRKY* members against various biotic and abiotic stresses in wheat.

## Data Availability Statement

The original contributions presented in the study are publicly available. This data can be found here: National Genomics Data Center, accession number: CRA003799 (https://bigd.big.ac.cn/bioproject/browse/PRJCA004312).

## Author Contributions

YW and JB designed the research. HY, LQ, HG, LG, and FR performed the experiments and analyzed the data. YW and HY wrote the manuscript. All authors reviewed and approved the final manuscript.

## Conflict of Interest

The authors declare that the research was conducted in the absence of any commercial or financial relationships that could be construed as a potential conflict of interest.

## Footnotes

1 https://urgi.versailles.inra.fr/download/iwgsc/IWGSC_RefSeq_Annotations/v1.0/

2 https://pfam.xfam.org

3 http://hmmer.org/download.html

4 https://www.ncbi.nlm.nih.gov/Structure/cdd/wrpsb.cgi

5 http://expasy.hcuge.ch/swissmod/SWISS-MODEL.html

6 https://gsds.cbi.pku.edu.ch

7 http://gsds.cbi.pku.edu.cn/

8 https://www.wheat-expression.com/

9 https://www.arabidopsis.org/

10 https://www.genome.jp/kegg/pathway.html

11 https://bigd.big.ac.cn

## Supplementary Material

Supplementary Figure 1 Analysis of the exon–intron structure of *TaWRKY* genes.

Supplementary Figure 2 Analysis of the conserved motifs of *TaWRKY* genes. The protein sequence of each motif was also indicated.

Supplementary Figure 3 Chromosomal locations of *TaWRKY* genes.

Supplementary Figure 4 Classification of TaWRKY proteins. The typical WRKY proteins from *Arabidopsis* are used as standards. A phylogenetic tree is constructed using N-J method in MEGA 6.0 software. **(A)** The TaWRKY proteins on chromosome 1–7 are classified orderly. **(B)** Some TaWRKY proteins that have long protein sequences are classified specially.

Supplementary Figure 5 The expression heat map of all *TaWRKY* gene copies. The expression data are downloaded from International Wheat Genome Sequencing Consortium. The candidate genes which can respond to abiotic stresses are highlighted by blue color. The expression levels are normalized using the log_2_ tpm values.

Supplementary Figure 6 Isolation of positive transgenic *Arabidopsis* lines. **(A)** RT-PCR confirmation of positive transgenic *Arabidopsis* lines. The *Arabidopsis ACTIN2* gene is used as inner reference. Wild type (col-0) is used as the negative control. **(B)** Green fluorescent protein (GFP) signal detection of positive transgenic *Arabidopsis* lines.

Supplementary Figure 7 Statistics of seed germination rate and primary root length of wild type (WT) and OE lines under normal condition. **(A)** Observations of seed germination status of WT and OE lines at 7 days after germination. **(B)** Statistics of seed germination rate of WT and OE lines at 7 days after germination. One-way ANOVA, followed by Tukey honest significant difference (HSD) test, was performed. There are no significant differences between WT and each OE line. Bars show means ± SD. This experiment is replicated five times. **(C)** Observations of primary root elongations of WT and OE lines at 7 days after germination. Scale bars: 1 cm. **(D)** Statistics of primary root elongations of WT and OE lines at 7 days after germination. One-way ANOVA, followed by Tukey HSD test, was performed. There are no significant differences between WT and each OE line. Bars show means ± SD, *n* = 20. This experiment is replicated five times.

Supplementary Figure 8 Gene Ontology (GO) and Kyoto Encyclopedia of Genes and Genomes (KEGG) analyses of differentially expressed genes (DEGs) isolated from polyethylene glycol (PEG) and salt stress, respectively. **(A)** GO term analysis of DEGs identified from PEG treatment. **(B)** KEGG enrichment of DEGs identified from PEG treatment. **(C)** GO term analysis of DEGs identified from salt treatment. **(D)** KEGG enrichment of DEGs identified from salt treatment.

Supplementary Figure 9 Gene Ontology term analysis of seven common differentially expressed genes isolated from polyethylene glycol and salt treatments.

Supplementary Table 1 The primers used in this study.

Supplementary Table 2 The *p*-value of each *TaWRKY* gene based on the results of hmmsearch.

Supplementary Table 3 Information of all *TaWRKY* genes that were isolated in this study.

Supplementary Table 4 The expression profiles of *TaWRKY* genes under different stresses based on the expression data from the database.

Supplementary Table 5 Differentially expressed gene list of each Gene Ontology term that the seven common genes took part in.
