# Supplementary figures and images for: Genome-Wide Identification of Wheat WRKY Gene Family Reveals That TaWRKY75-A Is Referred to Drought and Salt Resistances
Source: Front Plant Sci. 2021 Jun 4;12:663118. doi: 10.3389/fpls.2021.663118 (PMC8212938; doi:10.3389/fpls.2021.663118)

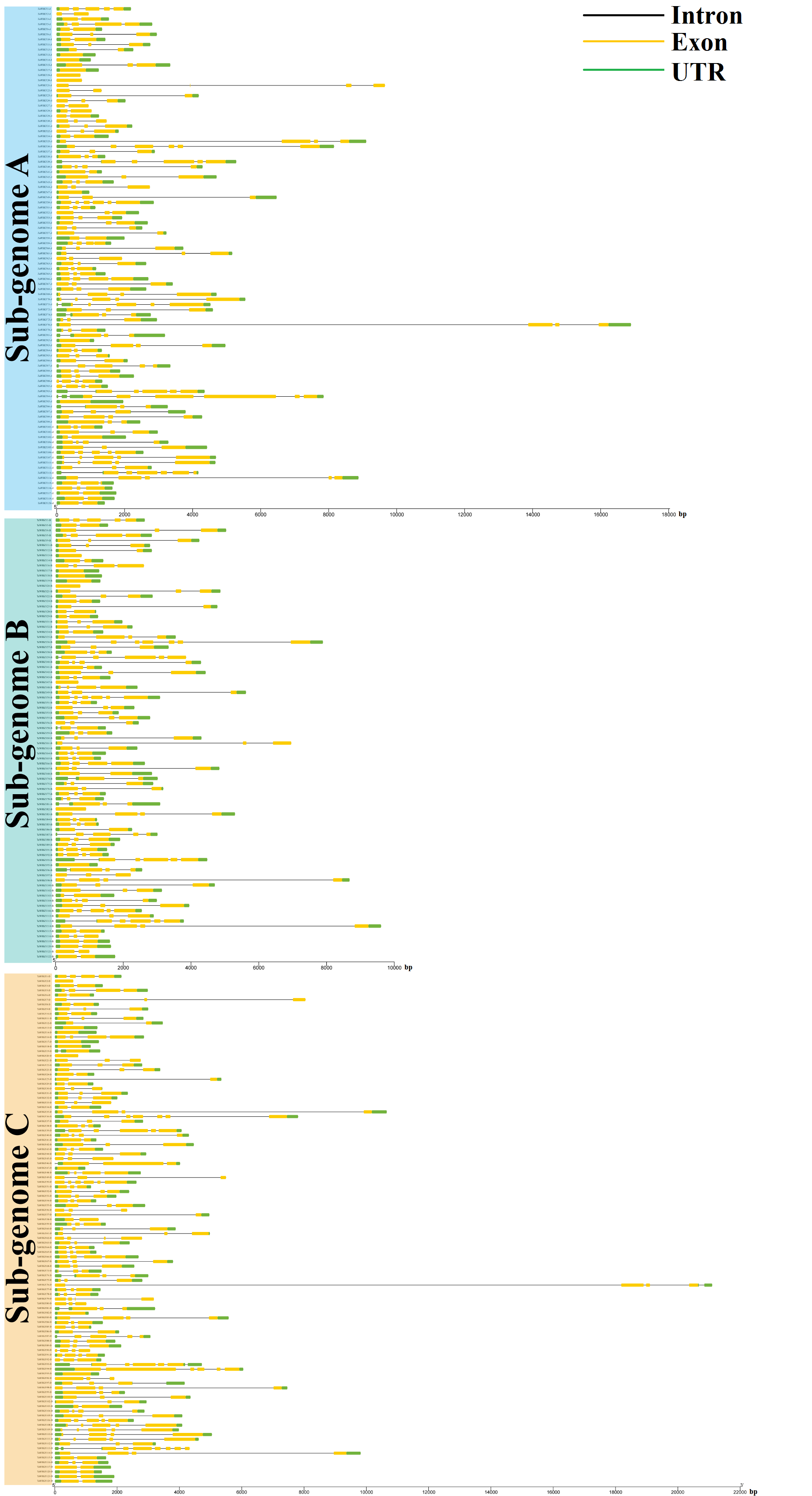

Supplement: Supplementary Figure 1 — Analysis of the exon–intron structure of TaWRKY genes. [file Image_1.TIF]

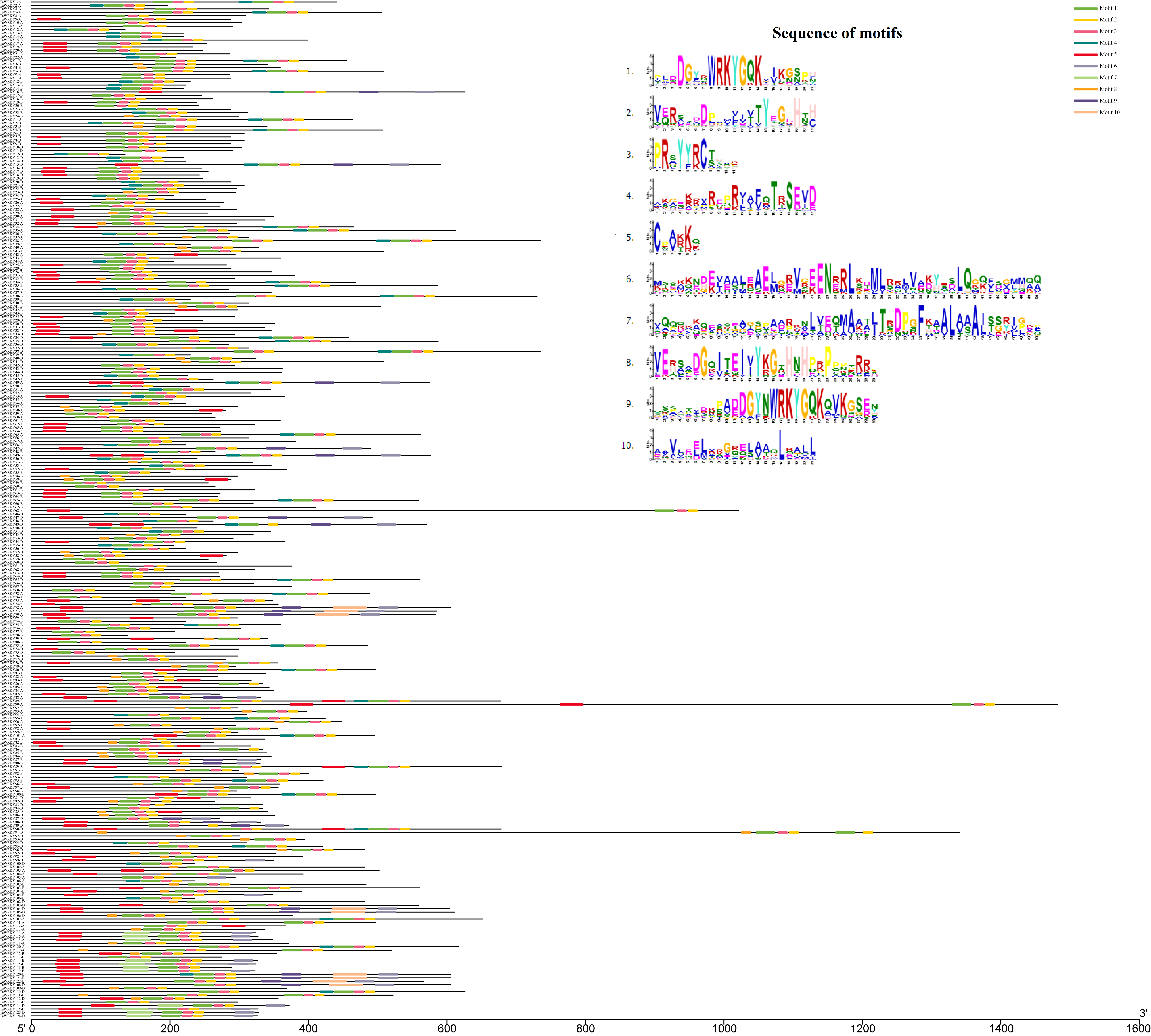

Supplement: Supplementary Figure 2 — Analysis of the conserved motifs of TaWRKY genes. The protein sequence of each motif was also indicated. [file Image_2.TIF]

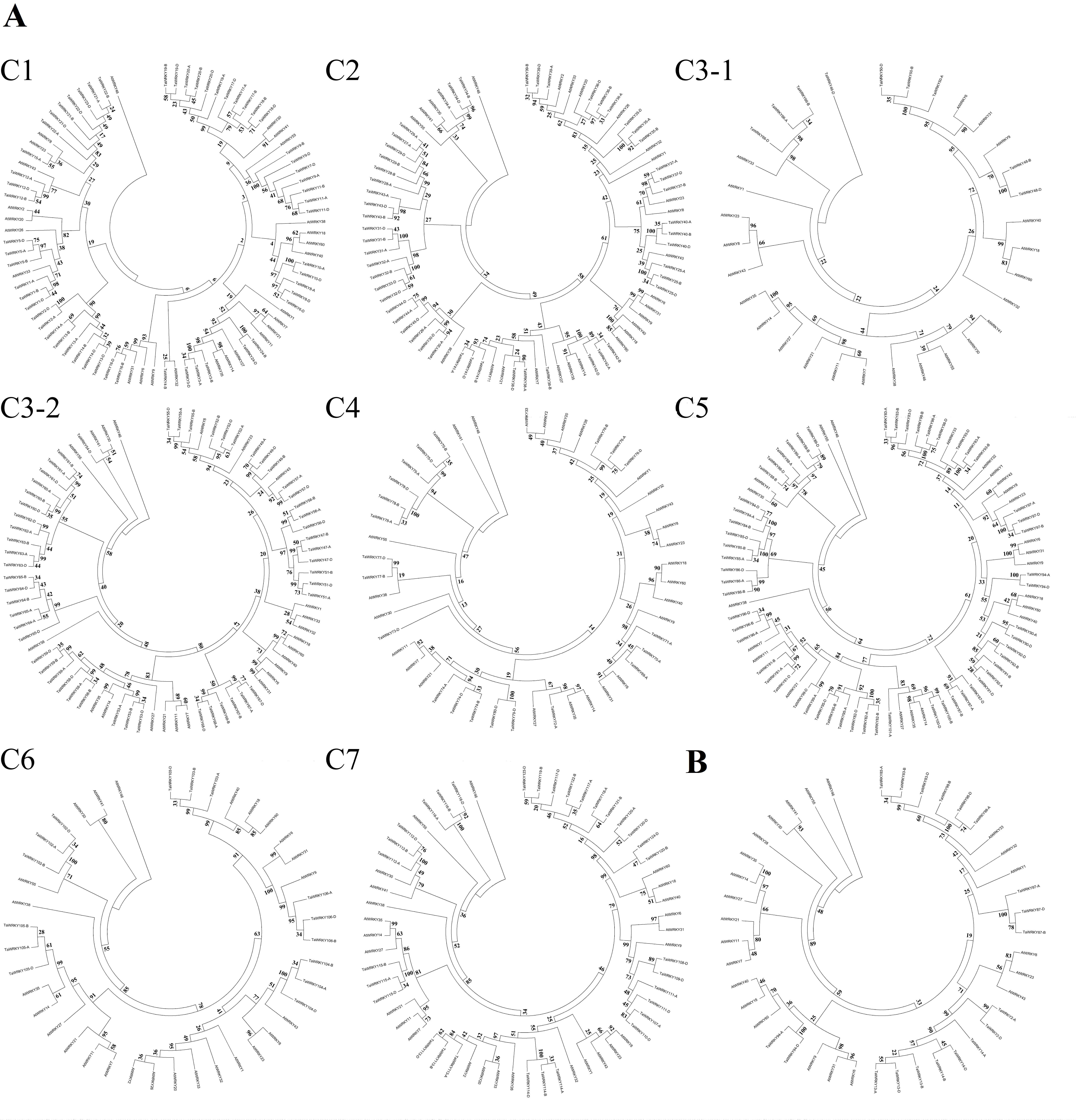

Supplement: Supplementary Figure 4 — Classification of TaWRKY proteins. The typical WRKY proteins from Arabidopsis are used as standards. A phylogenetic tree is constructed using N-J method in MEGA 6.0 software. (A) The TaWRKY proteins on chromosome 1–7 are classified orderly. (B) Some TaWRKY proteins that have long protein sequences are classified specially. [file Image_4.TIF]

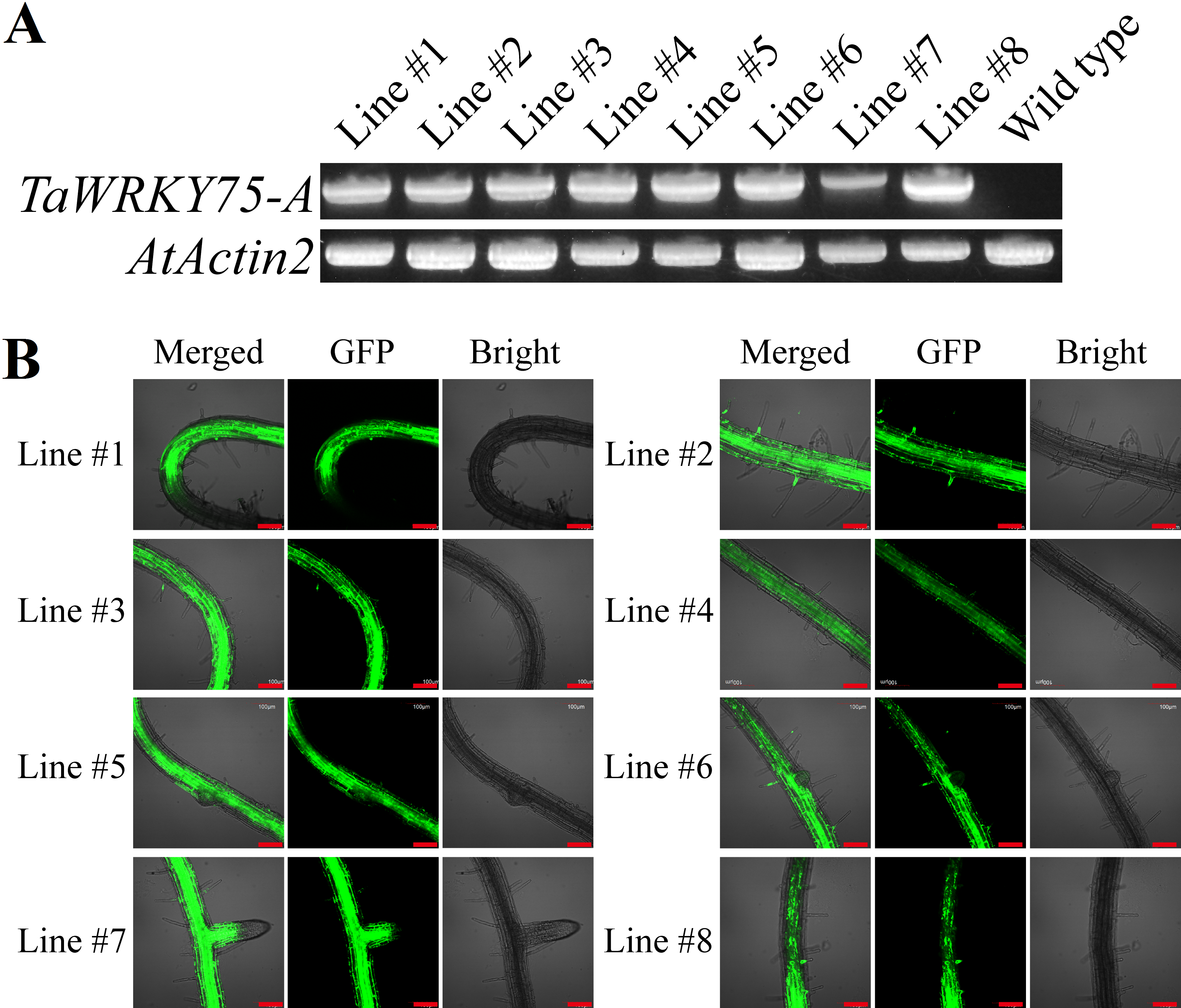

Supplement: Supplementary Figure 6 — Isolation of positive transgenic Arabidopsis lines. (A) RT-PCR confirmation of positive transgenic Arabidopsis lines. The Arabidopsis ACTIN2 gene is used as inner reference. Wild type (col-0) is used as the negative control. (B) Green fluorescent protein (GFP) signal detection of positive transgenic Arabidopsis lines. [file Image_6.TIF]

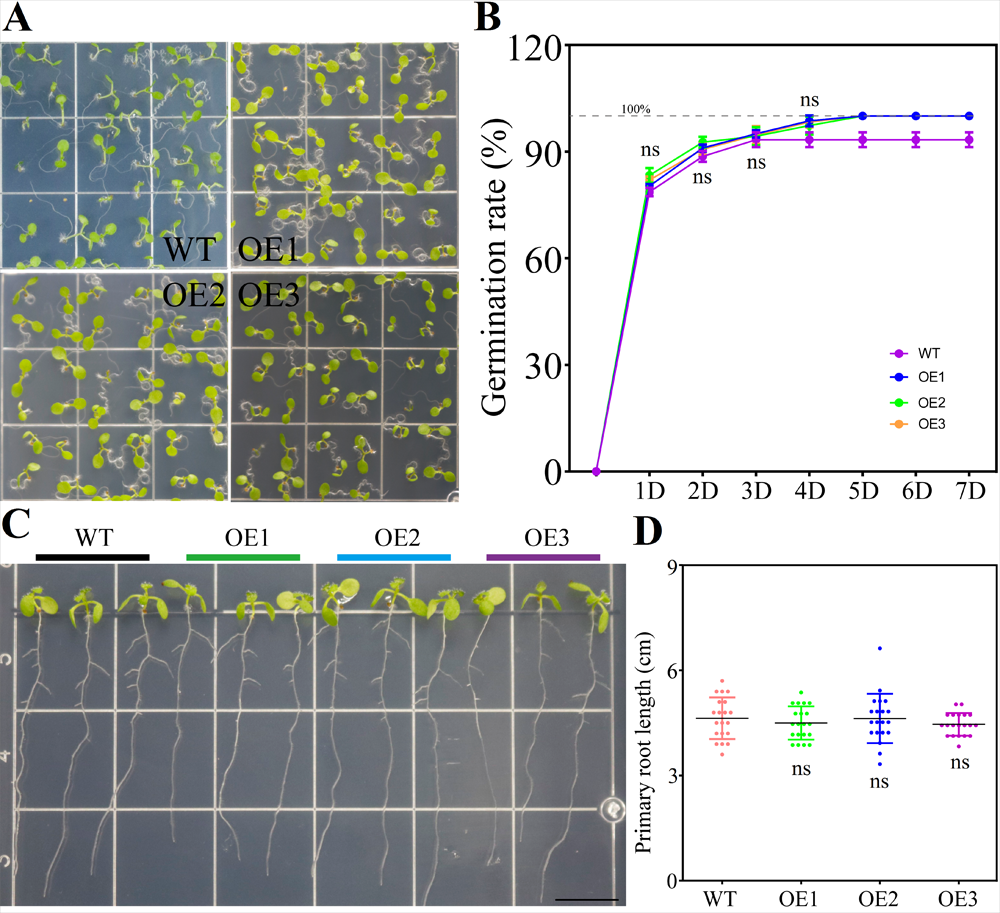

Supplement: Supplementary Figure 7 — Statistics of seed germination rate and primary root length of wild type (WT) and OE lines under normal condition. (A) Observations of seed germination status of WT and OE lines at 7 days after germination. (B) Statistics of seed germination rate of WT and OE lines at 7 days after germination. One-way ANOVA, followed by Tukey honest significant difference (HSD) test, was performed. There are no significant differences between WT and each OE line. Bars show means ± SD. This experiment is replicated five times. (C) Observations of primary root elongations of WT and OE lines at 7 days after germination. Scale bars: 1 cm. (D) Statistics of primary root elongations of WT and OE lines at 7 days after germination. One-way ANOVA, followed by Tukey HSD test, was performed. There are no significant differences between WT and each OE line. Bars show means ± SD, n = 20. This experiment is replicated five times. [file Image_7.TIF]

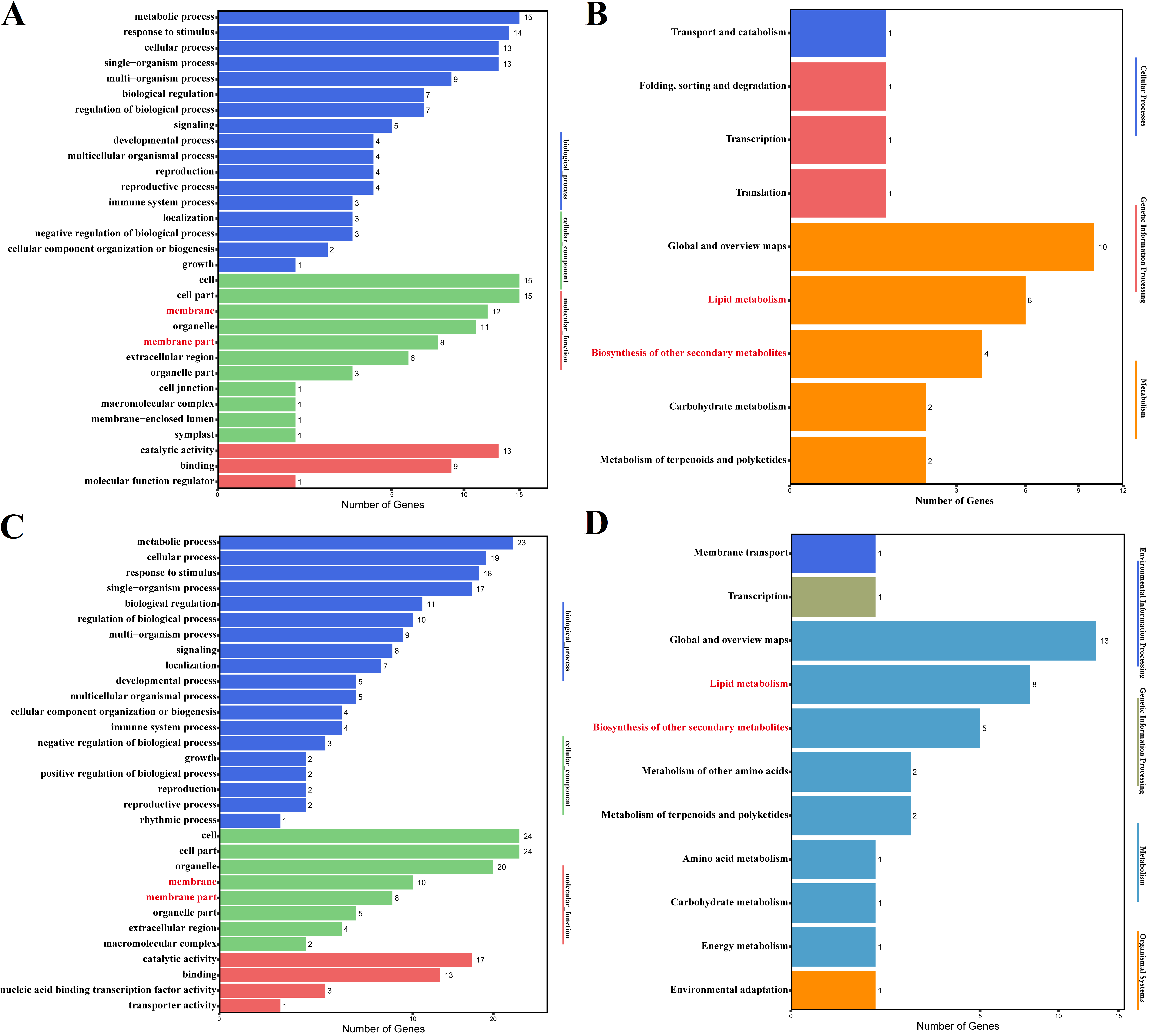

Supplement: Supplementary Figure 8 — Gene Ontology (GO) and Kyoto Encyclopedia of Genes and Genomes (KEGG) analyses of differentially expressed genes (DEGs) isolated from polyethylene glycol (PEG) and salt stress, respectively. (A) GO term analysis of DEGs identified from PEG treatment. (B) KEGG enrichment of DEGs identified from PEG treatment. (C) GO term analysis of DEGs identified from salt treatment. (D) KEGG enrichment of DEGs identified from salt treatment. [file Image_8.TIF]

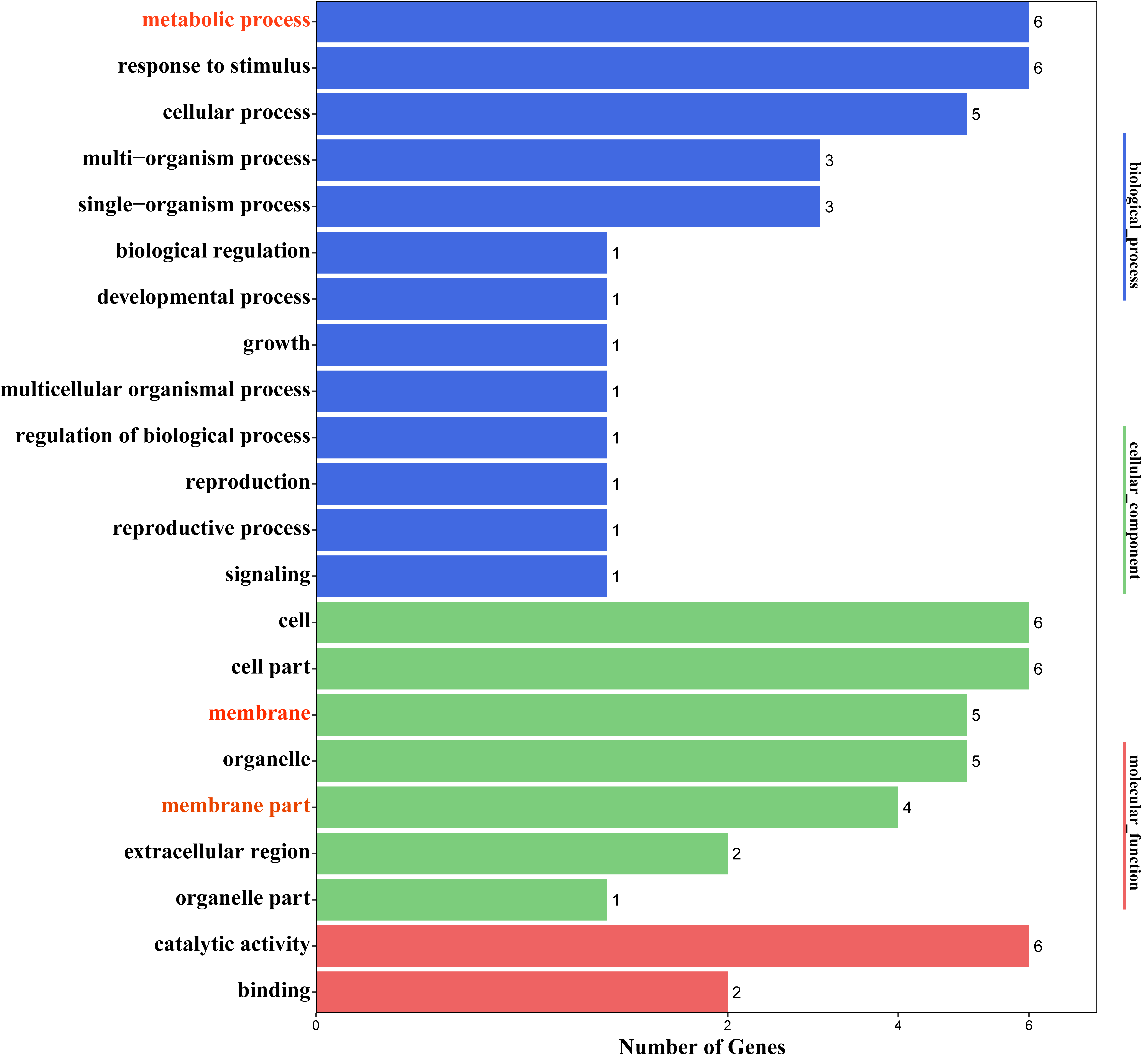

Supplement: Supplementary Figure 9 — Gene Ontology term analysis of seven common differentially expressed genes isolated from polyethylene glycol and salt treatments. [file Image_9.TIF]
